# Analysis for bearing performance and construction mechanical behavior of supporting structure for the large cross-section tunnel by half bench CD method

**DOI:** 10.1371/journal.pone.0255511

**Published:** 2021-08-06

**Authors:** Rui Zhao

**Affiliations:** School of Civil Engineering, Weinan Vocational and Technical College, Weinan, Shaanxi, China; China University of Mining and Technology, CHINA

## Abstract

Based on the engineering practice of large cross-section highway tunnel, this paper reveals the space-time coordinated evolution law of the construction mechanical characteristics and deformation distribution of the support structure in the construction by half bench CD method through field test. At the same time, the mechanical response calculation model of the supporting structure in the partial excavation is constructed, and the mechanical characteristics of the support structure in the partial excavation process are analyzed by above mechanical calculation model. Then, the mechanical and deformation distribution of the feet-reinforcement bolt in the steel frame—foot-reinforcement bolt combined support system is analyzed under different levels of surrounding rock load and different structural parameters of the feet-reinforcement bolt. The research results show that: (1) The internal force of the supporting structure changes most obviously during the excavation of Part Ⅰ, Part Ⅱ and Part Ⅲ, and the internal force of the support structure gradually tends to be stable after a slight increase in the excavation of Part Ⅳ and Part Ⅴ; (2) The horizontal deformation and vertical deformation of the support structure mainly occur in the excavation process of Part Ⅰ, Part Ⅱ and Part Ⅲ, and the excavation of Part Ⅳ and Ⅴ has little effect on the deformation response of the structure. The vertical displacement of the supporting structure is larger than the horizontal displacement, and the dynamic response of the temporary diaphragm structure during tunnel excavation is shrinkage-expansion-shrinkage-expansion; (3) The bending strain of each measuring point decreases with the increase of the distance from the loading point, and the bending strain of section 1 and section 2 is much larger than that of the other three sections; (4) With the increase of the angle, the section position with strain close to 0 gradually moves to the deeper position of the bolt, and the axial strain of each section on the bolt gradually changes from positive strain to negative strain.

## 1. Introduction

With the continuous development of China’s infrastructure construction, for the long-term consideration, the demand for the increase of driving width in the highway construction in many areas is very strong, which significantly increases the construction frequency of the large cross-section highway. tunnels. At present, there are neither mature experience nor specifications for reference [1–5]. During the construction of large cross-section tunnel, excavation and support structure installation are often carried out continuously. This dynamic construction mode makes it difficult to control the safety of the support structure [6–8].

At present, the selection of construction method for large cross-section highway tunnel is mainly based on the surrounding rock conditions. For tunnel with weak surrounding rock, in order to ensure the stability of surrounding rock and supporting structure in the construction process, it is inevitable to adopt the partial excavation methods, such as cross diaphragm method (CRD) [9, 10], the two-side pilot hole method [11] and center diaphragm method (CD) [12]. The excavation process in these methods is complicated and the efficiency is relatively low. The relationship between the construction efficiency and the structure safety has become one of the main contradictions in the construction of large cross-section tunnel. Therefore, it is urgent to seek a rapid construction method that can meet the needs of tunnel safety and construction efficiency. Through the gradual improvement and optimization of engineering practice, based on the traditional CD method, the half bench CD method is formed. The new construction method not only simplifies the construction steps, but also effectively ensures the safety and stability of the tunnel structure in the construction process, and realizes the synchronous improvement of the safety and economy of tunnel engineering. With the gradual improvement of structural safety requirements in tunnel engineering practice, the mechanical response and deformation behavior of support structure in the excavation process are particularly important for the safety and stability of the whole structure, and gradually become the engineering difficulties in tunnel construction and the research focus of many experts and scholars [13–16].

Zhang et al. studied the stress of the temporary middle wall in the construction process of CRD method by field monitoring and numerical simulation, and obtained the rules that the internal force of the middle wall increased during the whole excavation process, and the safety factor reached the minimum after the excavation of Part Ⅳ [17]. Sun et al. applied numerical simulation and field monitoring data to analyze the mechanical characteristics of the middle partition wall in the construction of the two side pilot hole method, and proposed that after tunnel excavation, the main stress and bending moment of the middle partition wall basically distribute symmetrically along the tunnel cross-section center line, the maximum tensile stress appears on the middle partition wall, and the maximum compressive stress appears at the intersection of the middle partition wall and the middle partition wall [18]. Hou and others studied the dynamic response of the middle wall structure during the tunnel blasting construction through the dynamic damage test of the middle wall and ANSYS / LS-DYNA, and proposed that the middle wall is in the repeated tension and compression state under the action of blasting stress wave, and the center, top and bottom positions of the middle wall support structure are the part of stress concentration and the most vulnerable parts of the structure [19]. By establishing a mechanical model, Zhang et al. analyzed the mechanical characteristics of the primary support structure and the temporary middle wall structure in the construction of the two-side pilot hole method. In his analysis, the side wall steel frame was regarded as an arch structure with both ends fixed in the stratum, and the middle wall structure was regarded as a beam structure with both ends fixed in the stratum [20]. The moment transfer and deformation coordination between the side wall steel frame and the middle wall structure were not considered. The stress form of supporting structure is quite different from the actual situation. Luo et al. based on the super long span highway tunnel, established the mechanical calculation model of supporting structure with bearing and deforming together in partial excavation, and obtained that in the process of tunnel excavation, the axial force of steel frame fluctuates repeatedly with the excavation of different stages, especially the stress at the middle wall steel frame changes most significantly, and the stress of the whole structure gradually tends to be stable after the installation of the tunnel lining [21]. The surrounding rock of the above research project is relatively good and the tunnel excavation height is large. Therefore, in the model construction, the middle partition wall structure is equivalent to a straight beam, the foot lock bolt is considered to bear the all-upper load, and the bearing effect of the foundation soil at the bottom of the steel frame is not considered. However, when the surrounding rock of the tunnel is weak and the influence of excavation span on the shape of the diaphragm structure cannot be ignored, the load of the steel frame structure should be shared by the foot lock bolt and the foundation soil. At the same time, the influence of the curvature of the diaphragm structure on the overall mechanical bearing capacity of the supporting structure should be considered in the construction of the model.

The selection of construction method for large cross-section tunnel is mainly based on the surrounding rock conditions [22–26]. At present, the research on the construction mechanical behavior of the support structure for large cross-section tunnels using the partial excavation method mainly focuses on the traditional construction methods such as CD, CRD and the two-side pilot hole method, and there is little research on the half bench CD method which can improve the construction efficiency and save the cost of project. At the same time, the previous researches mainly focus on the comparative analysis and applicability analysis between different construction methods [27–29], and seldom collaboratively consider the mechanical characteristics and deformation laws of the supporting structure, which is very important for the safety and stability and dynamic control of the tunnel construction. In the past, numerical simulation is often used in the complicated excavation process [30–34]. However, the difficulty of obtaining the simulation parameters accurately and the large difference between the model and the engineering practice made the simulation results often differ from the actual situation. At the same time, in the few mechanical model calculation studies, the calculation model is not reasonably constructed according to the actual situation of tunnel excavation. In addition, many assumptions in the previous calculation models make the mechanical analysis model deviate greatly from the actual support situation of the tunnel in engineering practice.

This paper, based on the engineering practice of large cross-section highway tunnel, aiming at the construction mechanical response of the support structure in the application of half bench CD method, reveals the space-time coordinated evolution law of the construction mechanical characteristics and deformation distribution of the support structure through field test. At the same time, according to the construction mechanical behavior and the dynamic response mechanism of the primary support steel frame and the temporary middle wall structure during the tunnel excavation, the mechanical response calculation model of the primary support structure in the partial excavation by half bench CD method is constructed, and the mechanical characteristics of the support structure in the partial excavation process are analyzed by the above mechanical calculation model, which further verifies the engineering applicability of calculation method. Then, the mechanical and deformation distribution of the foot-reinforcement bolt are analyzed under different levels of surrounding rock pressure and different structural parameters of the foot-reinforcement bolt.

## 2. Engineering background and field test

**Fig 1:**
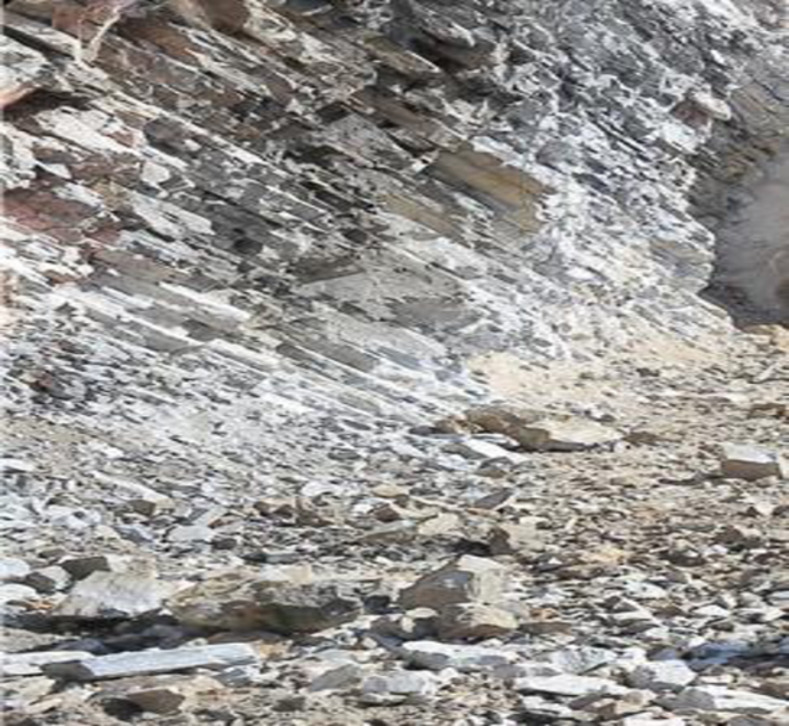
The surrounding rock characteristic in the tunnel site.

**Fig 2:**
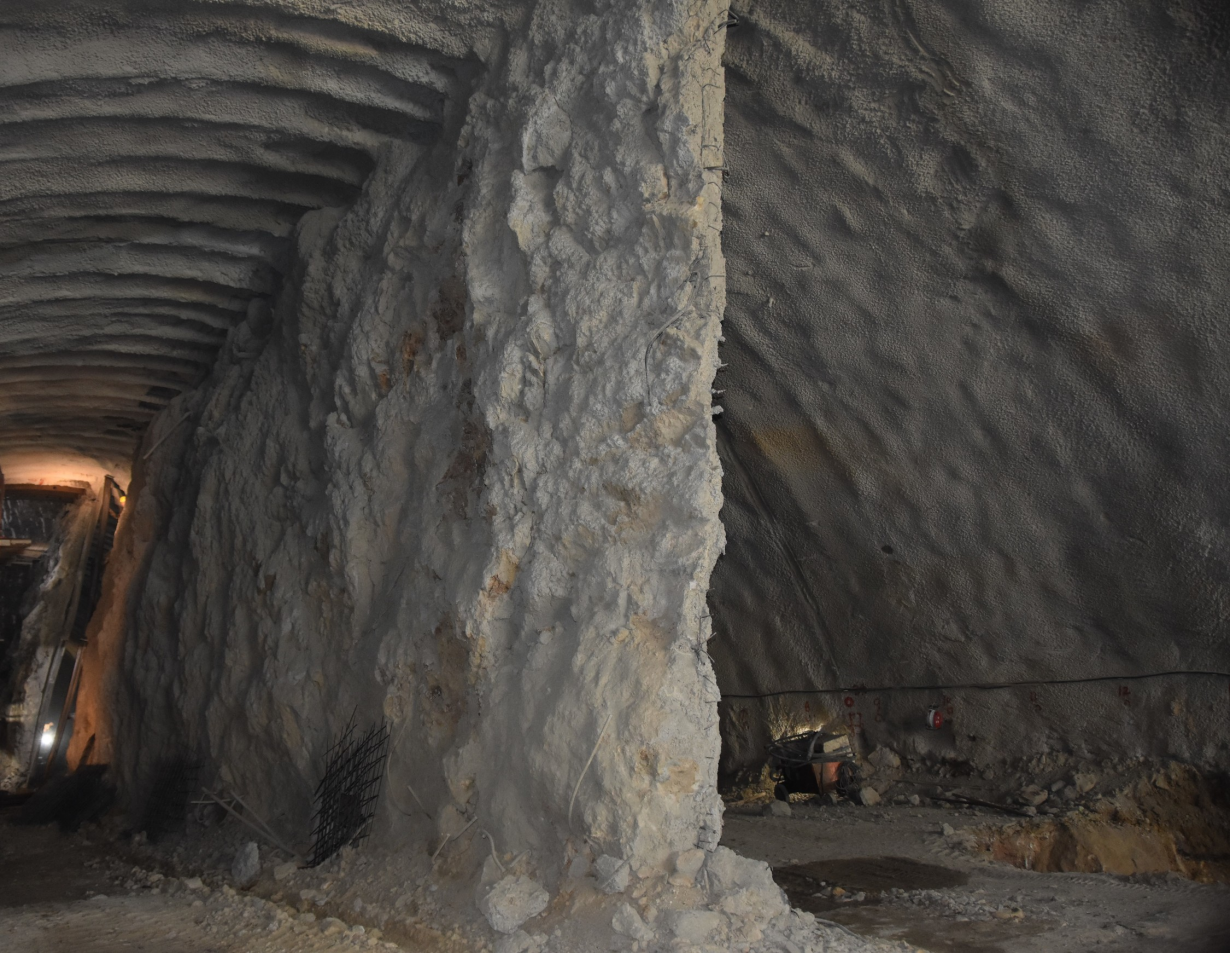
The construction of middle wall structure.

**Fig 3:**
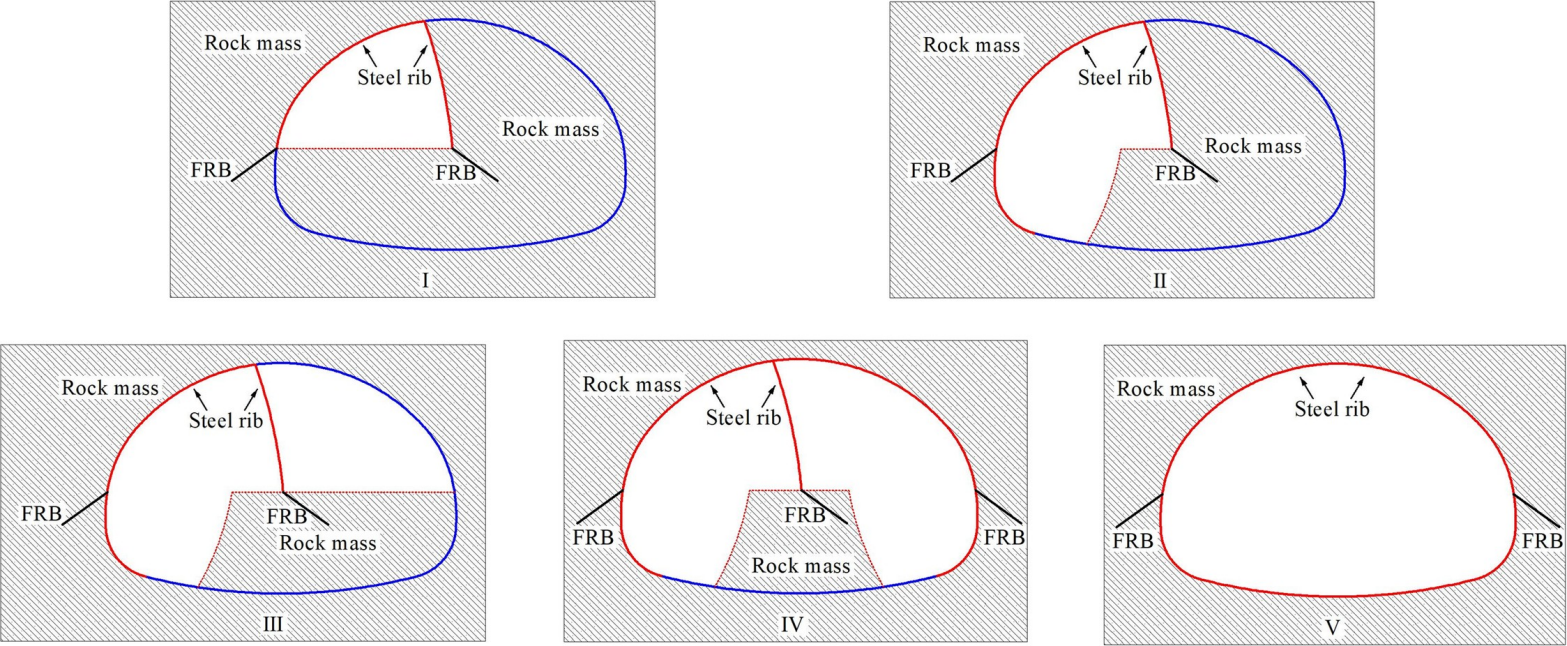
The main construction process of half bench CD method.

The project is located at the junction area of Yunnan Plateau and Hengduan Mountains, with the overall terrain of high in the northwest and low in the southeast. The terrain along the tunnel is complex with obvious undulation and high density of rock mass. The stratum in the project area is quaternary slope residual soil, and the surrounding rock is moderately weathered limestone and slate interlayer, which is strictly controlled by geological structure. The surrounding rock characteristic in the tunnel site is shown in Fig 1. In addition, the maximum excavation span of the tunnel is 20.96m, which belongs to large cross-section highway tunnel. According to the results of field geological survey, the surrounding rock grade belongs to grade IV. According to the characteristics of surrounding rock, on the basis of CD method and bench method, and taking advantage of the strong bearing capacity of basement surrounding rock, the temporary vertical support system is composed of the middle wall structure in upper bench and core rock reserved in lower bench. The specific construction processes are as follows: (1) The tunnel cross-section is divided into two benches, a total of five parts for excavation. The upper bench is supported by temporary middle wall, and the excavation is staggered left and right part, which reduces the excavation span effectively. (2) The core rock at the left and right side-walls of the lower bench is excavated step by step. After the installation of the primary support structure, the foot-reinforcement bolt is used to support in time to ensure the structural stability. Then the integral excavation support system constituted by the reserved core rock in the lower bench and the temporary middle wall structure in the upper bench effectively control the tunnel deformation. (3) Finally, the temporary middle wall structure is removed and the core rock in the lower bench is excavated, and the primary support structure is closed into a ring. Compared with the traditional CD method, this method reduces the procedures of temporary support installation and demolition, optimizes the construction sequence, increases the construction speed and reduces the project cost. The construction of middle wall structure and the main construction process are shown in Figs 2 and 3 respectively.

## 3. Analysis of field experiment results

**Fig 4:**
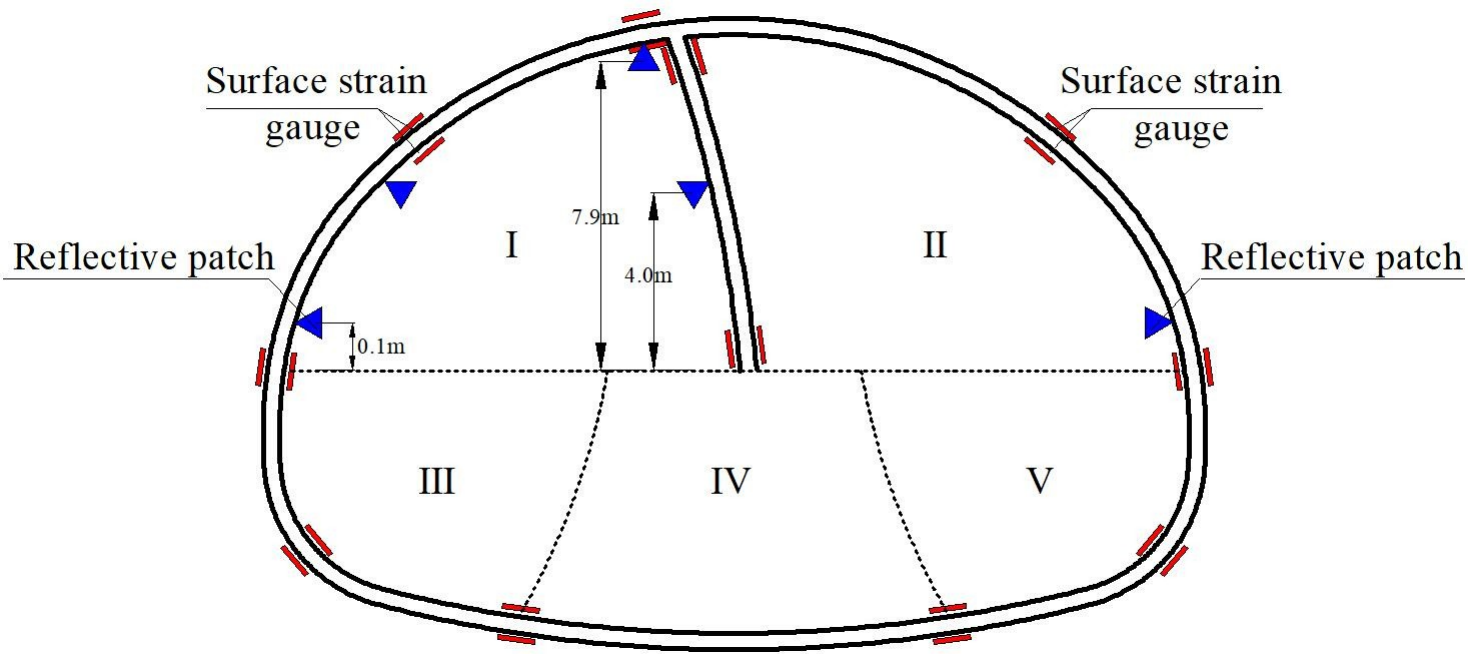
The layouts of the measuring points.

**Fig 5:**
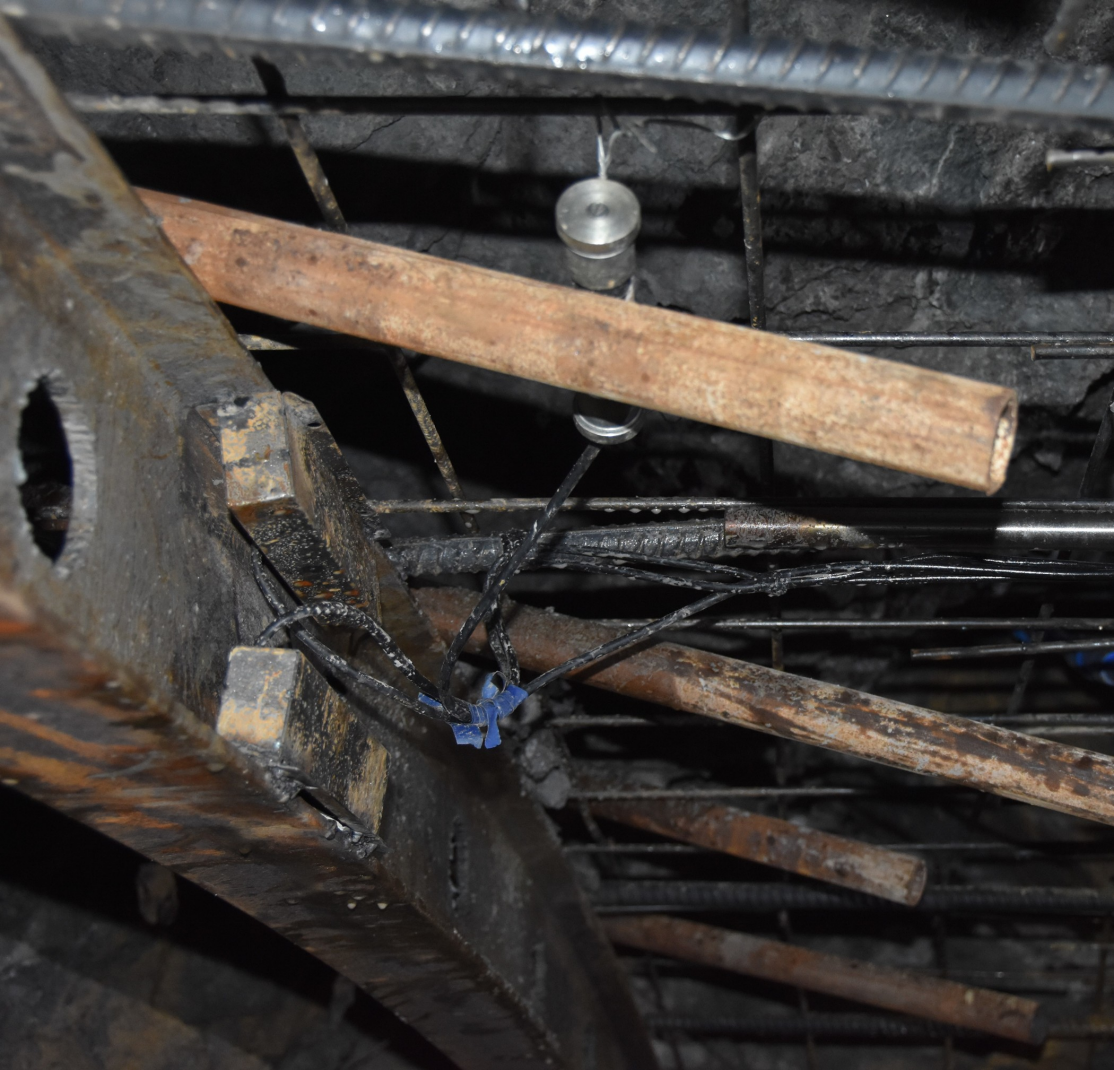
The site layouts of the strain gauge.

**Fig 6:**
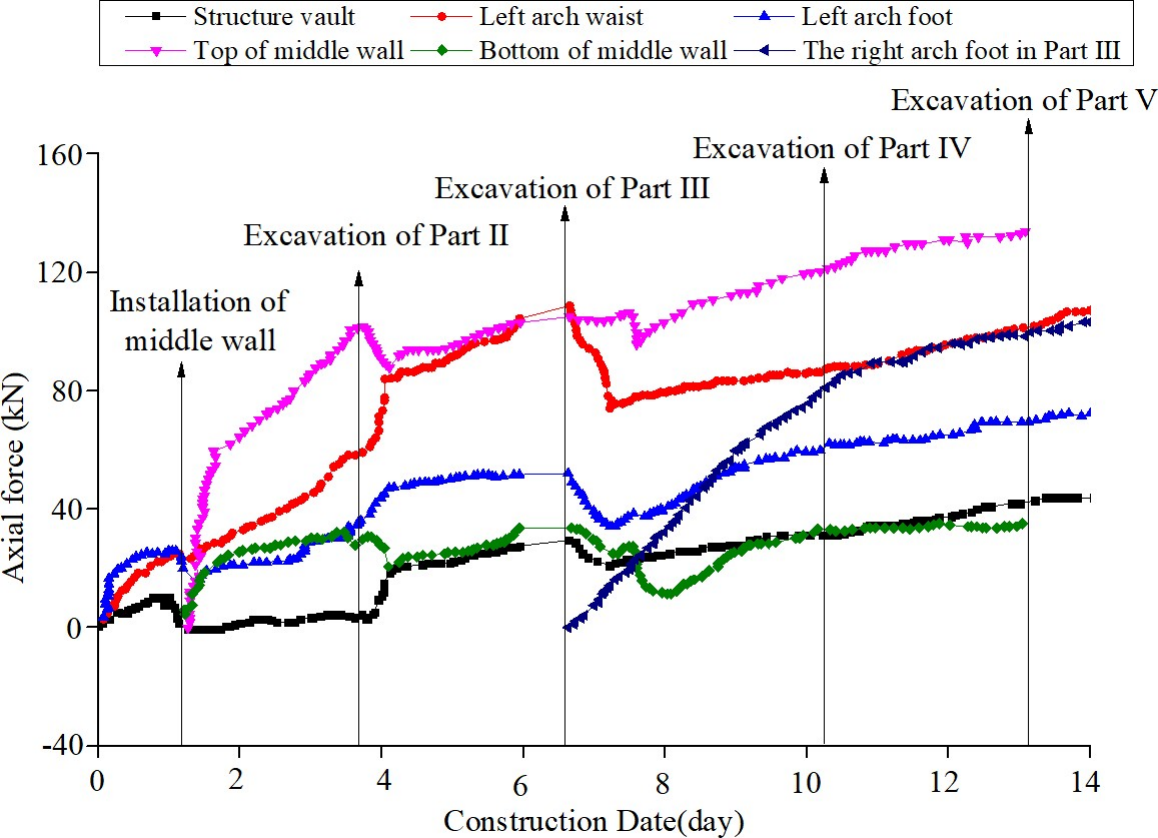
Axial force distribution of supporting structures in the tunnel excavation.

**Fig 7:**
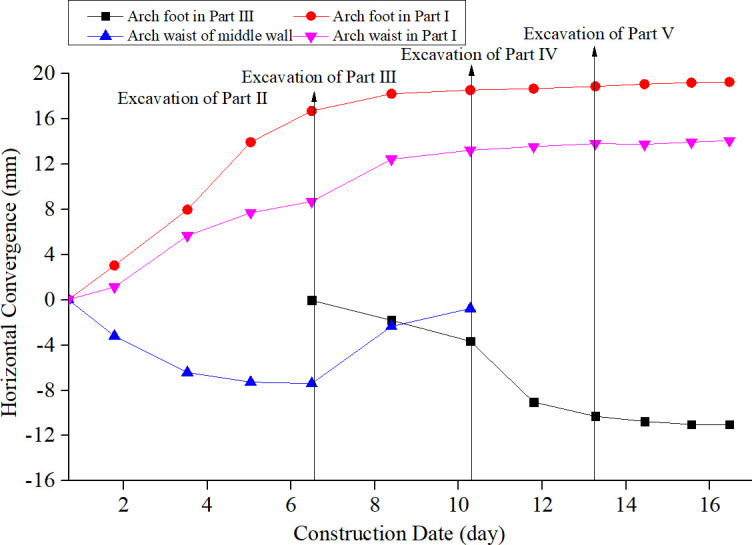
Horizontal convergence of support structure during tunnel construction.

**Fig 8:**
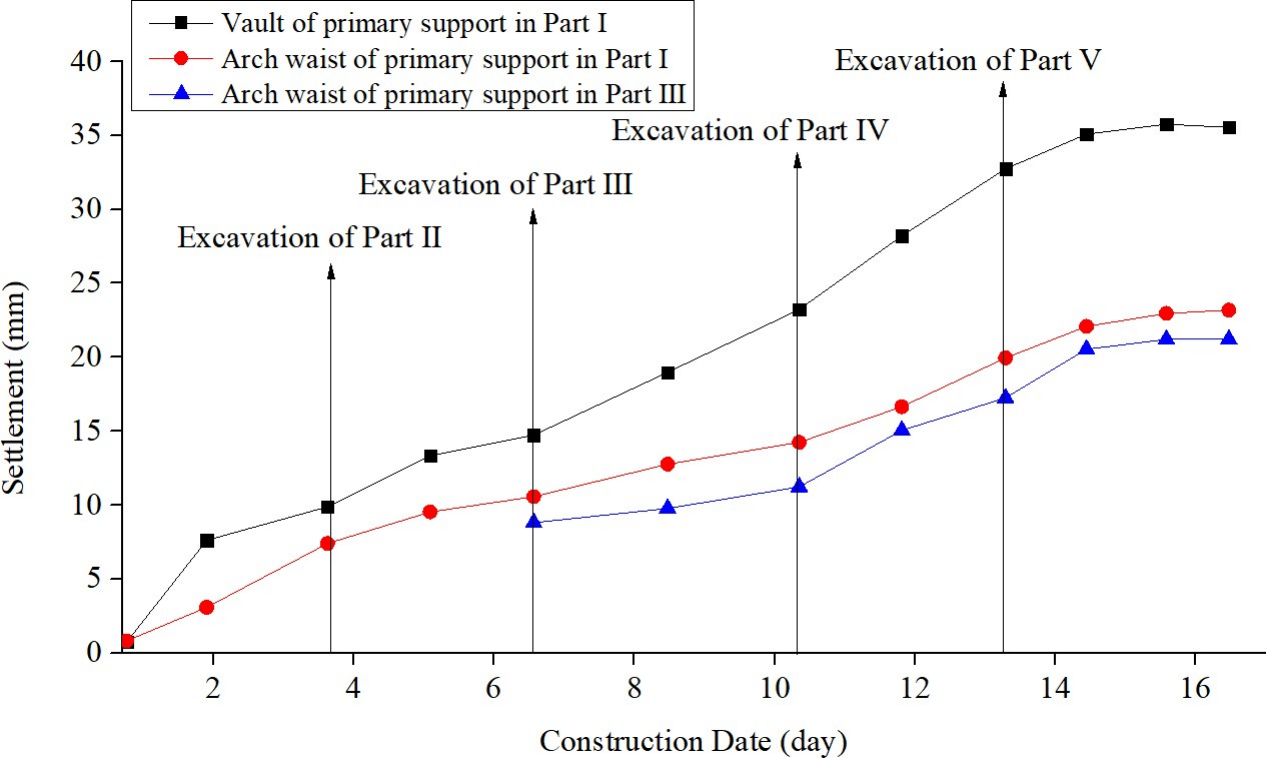
Settlement of support structure during tunnel construction.

This field test has been approved and supported by the Project Management Bureau. In order to study the construction mechanical behavior of the support structure by half bench CD method, the deformation and the stress of the supporting structures in each construction stage were monitored. The total station was used to monitor the horizontal deformation and vertical settlement of the key parts of the supporting structure during the tunnel excavation. The layouts of the measuring points are shown in Fig 4. The internal force of the tunnel supporting structure was monitored by the steel string surface strain gauge installed inside and outside the tunnel steel frame. The site layouts of the strain gauge are shown in Fig 5. And the results are shown in Figs 6–8.

According to the field test results, the internal force of each position of the supporting structure changes gradually with the excavation of each part. The maximum internal force of the structure appears at the bottom of the middle wall structure, and structure internal force increases rapidly after the installation of middle wall. During the excavation of each part, the excavations of Parts Ⅰ, Ⅱ and Ⅲ have a great influence on the internal force of the supporting structure, and the internal force value of each monitoring position in these three stages reaches 75% of the limit internal force of the whole excavation process. After the excavation of the Part Ⅰ, the primary support structure is installed immediately. At this time, the axial force at the vault, arch waist and arch foot of the primary support structure increases sharply. After the erection of the middle wall structure, the axial force at the top and bottom of the structure increases sharply. At this time, the axial force at each part of the primary support decreases slightly and then continues to increase. This is mainly because the support structure forms an integral bearing structure after the installation of the middle wall structure, and the load of the primary support structure is partially shared by the middle wall structure. In the short term, due to the readjustment of the stress, the internal force of the primary support reduces to a certain extent. After the structure reaches a stable bearing state, the surrounding rock pressure adjusts to a new state, and the supporting structure reaches a new stress balance, so the internal force of the structure further increases. The change of axial force distribution of the structure at this stage is due to the change of the overall support form of the support structure and the readjustment of the stress. The axial force at the arch waist increases rapidly, which is the position with the strongest structural mechanical response. After the excavation of the Part Ⅱ, the axial forces of the vault, the arch waist and the arch foot at the primary support structure increase sharply, and the internal forces at the top and bottom of the middle wall structure decrease briefly and then continue to increase. After the excavation of the Part Ⅲ, due to the readjustment of the surrounding rock load on the structure, the overall internal force of the support structure decreases briefly and then increases slowly, and the internal force at the arch waist and arch foot of the primary support structure changes most obviously. Then, the internal force of the support structure gradually tends to be stable after a slight increase in the excavation of Parts Ⅳ and Ⅴ, and the overall internal force tends to be stable after the support structure closed into a ring. The results show that the internal force of the support structure changes most obviously during the excavation of Parts Ⅰ, Ⅱ and Ⅲ, and the above three excavation stages are the most unfavorable period for the whole support structure. Compared with other excavation stages, the subsequent excavation of Part Ⅳ and Part Ⅴ has a certain influence on the internal force of the support structure.

According to the monitoring, the deformation of different positions at the support structures changes gradually with the excavation of each part. On the whole, the vertical displacement of the support structure is larger than the horizontal displacement, and both of them show the characteristics of shrinking to the tunnel clearance. At the same time, the dynamic response of the temporary diaphragm structure during tunnel excavation is shrinkage-expansion-shrinkage-expansion. From the overall structure deformation distribution, the horizontal deformation and vertical deformation of the support structure mainly occur in the excavation process of Part Ⅰ, Ⅱ and Ⅲ, and the excavation of Parts Ⅳ and Ⅴ has little effect on the deformation response of the structure. Generally speaking, with the advance of tunnel construction, the support structure shows different degrees of deformation response, and finally tends to be stable. For the vertical displacement, the settlement of the vault and the arch waist of the primary support structure after the excavation of the Part Ⅰ has been increasing, especially the excavation of the Part Ⅲ has promoted the increase of the deformation in those two positions. All in all, the settlement caused by the excavation of Part Ⅰ and Ⅲ accounts for about 75% of the total settlement, which indicates that the settlement of vault and arch waist are mainly caused by the excavation of the upper bench, and the excavation influence of the lower bench is small. For the horizontal displacement, the left and right arch feet of the primary support show the same change law: the horizontal deformation of the two positions all increases sharply after the excavation of the lower parts, reaching the maximum deformation. The horizontal deformation of the structure caused by the excavation of the lower part accounts for almost 50% of the total deformation. In the later excavation stages, the horizontal convergence increases slightly and then tends to be stable. Finally, the deformation of the left and right arch feet are the same. The horizontal deformation of the middle wall structure presents the opposite laws. The horizontal deformation increases rapidly after the excavation of Part Ⅰ, and the excavation of the Part Ⅱ leads to the lateral expansion of the middle wall structure, resulting in the partial recovery of the horizontal deformation. After the excavation of the Part Ⅲ, the horizontal deformation of the middle wall structure expands further, and the structural deformation gradually tends to be stable during the subsequent stages. The horizontal deformation of the middle diaphragm structure mainly occurs in the excavation process of upper bench, and the excavation of this part has limited influence on the horizontal deformation of the primary support structure. The excavation of the rock mass at the lower bench is the main affecting factor for the horizontal deformation of the primary support arch foot.

## 4. Computational model of mechanical evolution

In the half bench CD excavation method for large cross-section tunnel, the excavation is mainly divided into upper and lower benches, a total of five parts for excavation. The upper bench is supported by temporary middle wall structure, and the left and right parts are excavated alternately. Then the middle core rock is reserved in the lower bench, and the rock mass at the left and right side-walls is excavated step by step. According to the field engineering practice analysis, it can be seen that the bottom of the primary support structure after the excavation of the rock mass in the lower side wall deformed greatly. Therefore, the bottom of the steel frame is firmly connected with the foot-reinforcement bolt to ensure the structure stability in time, and then the whole support system is formed by the core rock mass reserved in the lower bench and the temporary middle wall support of the upper bench to make sure that the deformation of the tunnel could be effectively controlled. Finally, the temporary middle wall support was removed and the core rock mass reserved at the lower bench was excavated. At this time, the whole supporting structure system closed into a ring.

**Fig 9:**
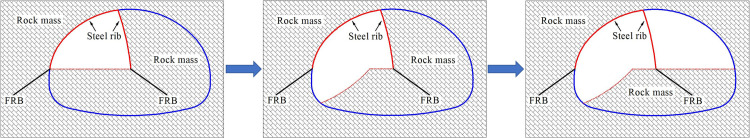
The excavation process of Part Ⅰ, Ⅱ and Ⅲ in tunnel.

**Fig 10:**
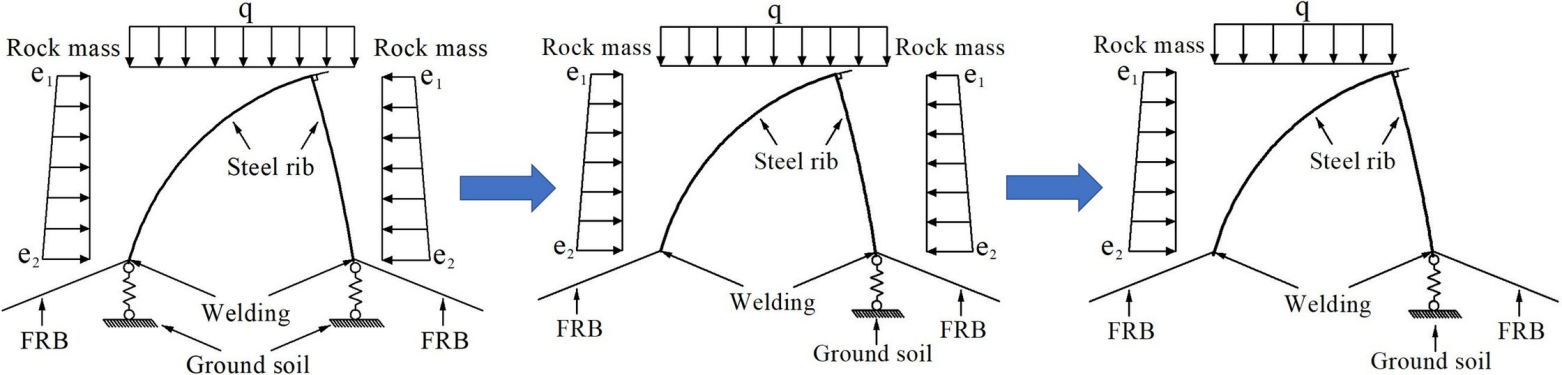
The bearing mode of support structure during tunnel excavation.

**Fig 11:**
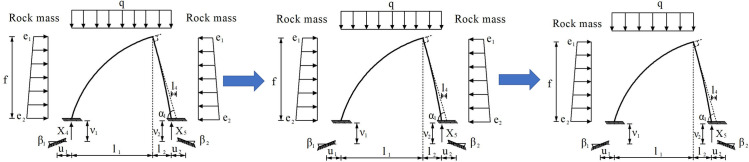
The mechanical evolution calculation model of support structure during excavation.

The excavation process of Part Ⅰ, Ⅱ and Ⅲ in tunnel is shown in Fig 9. The bearing mode and mechanical evolution calculation model of support structure during excavation is shown in Figs 10 and 11 respectively.

After the excavation of Part Ⅰ, the primary support structure and temporary middle wall structure are installed in time, and the bottom of the steel frames are welded firmly with foot-reinforcement bolt; After that, with the excavation of supporting rock in Part Ⅱ, the foundation rock mass at the lower part of the steel frame disappears, which means that the steel frame structure in Part Ⅰ is no longer affected by the bearing capacity of the foundation soil in Part Ⅱ; At this time, the excavation of the surrounding rock in Part Ⅲ makes the middle wall structure no longer bear the surrounding rock pressure from that part.

The model structure satisfies the following assumptions: 1. The primary support structure and temporary middle wall structure are regarded as the equal diameter arcs, both of which are vertically connected at the top position; 2. The inherent elastic support of the foundation rock mass to the steel frame structure is realized by the vertical elastic link set at the arch foot of the steel frame; 3. The supporting action of foundation rock accords with *Winkler* hypothesis; 4. After the foundation rock of the Part Ⅲ is excavated, the foot-reinforcement bolt at the bottom totally bears the load from the upper steel frame structure.

The vertical pressure of surrounding rock is assumed to be uniform load *q*. When the property of rock mass is poor, the horizontal pressure should be calculated according to the trapezoidal distribution load. *e*_1_ is the horizontal lateral pressure at the vault, and *e*_2_ is the horizontal lateral pressure at the arch foot. *f* is the excavation height (Height of steel frame). *β*_1_, *ν*_1_ and *μ*_1_ are the angle, vertical displacement and horizontal displacement of arch foot of the primary support structure respectively; *β*_2_, *ν*_2_ and *μ*_2_ are the angle, vertical displacement and horizontal displacement of arch foot of the temporary middle wall structure respectively; *α* is the angle between the tangent line of the top position of the middle wall structure and the horizontal direction; *l*_1_ and *l*_2_ are the horizontal projection length of the primary support structure and the middle wall structure respectively, *l*_4_ is the horizontal distance between the point formed by the intersection of the horizontal plane and the tangent line at the top of the middle wall structure and the arch foot of the middle wall structure; *X*_4_ and *X*_5_ are the supporting reaction provided by the foundation rock at the arch foot of the primary support structure and the middle wall structure respectively.

## 5. Mechanical calculation of model structure

**Fig 12:**
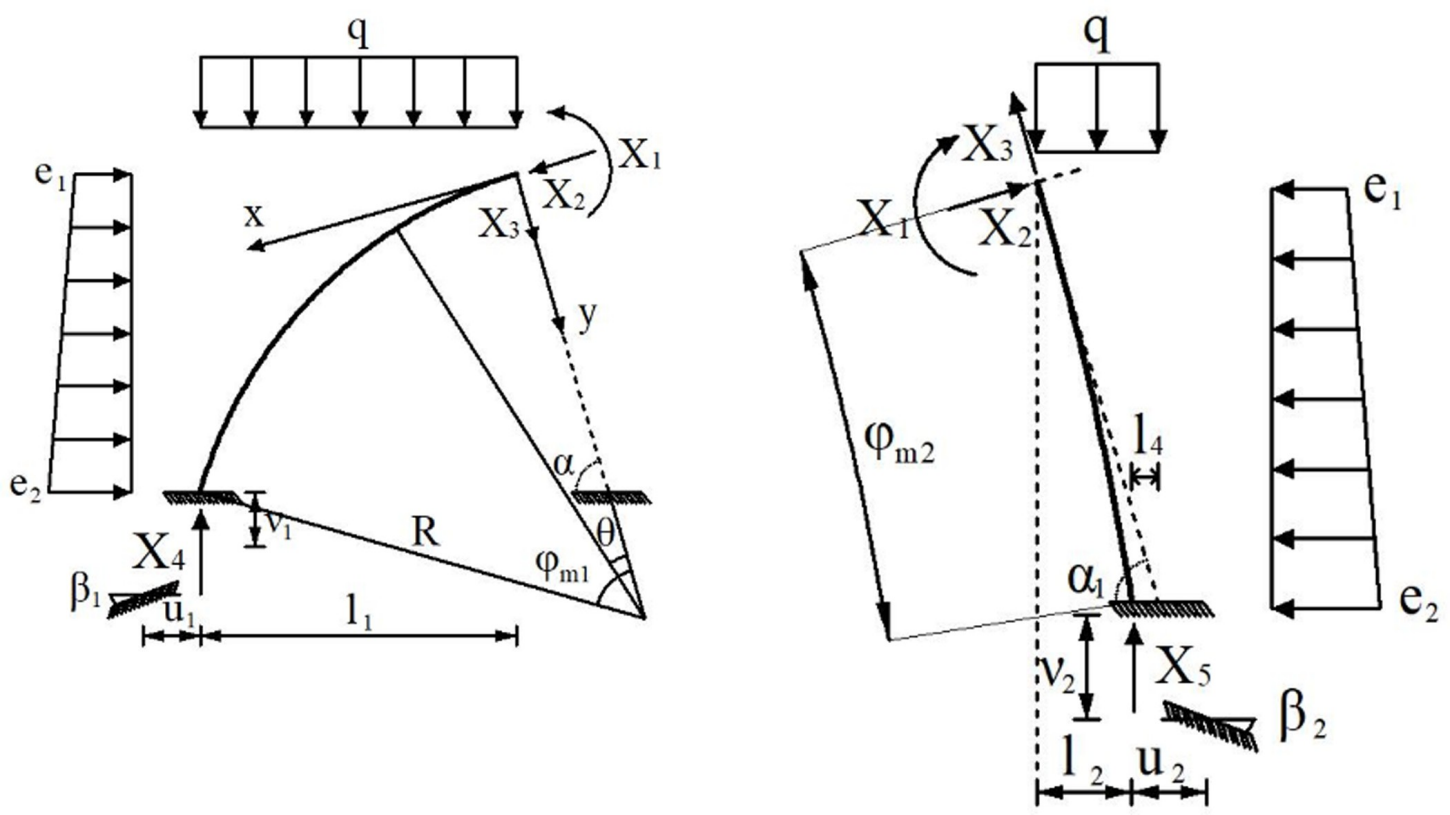
The basic mechanical calculation system of supporting structure. (a) Primary support, (b) Middle wall.

The internal force of support structure is calculated by force method. Dividing the whole support structure into two parts from the structure vault, three pairs of redundant unknown forces *X*_1_ (bending moment), *X*_2_ (axial force) and *X*_3_ (shear force) are used to represent the internal force of the structure at that position. The support reaction of the foundation rock at the arch foot of the primary support structure and the middle wall structure is replaced by *X*_4_ and *X*_5_ respectively. Because the whole supporting structure is asymmetry, the asymmetric elastic deformation occurs at the left and right steel frame arch feet, so the horizontal displacement *u*, vertical displacement *ν* and angle *β* of the arch feet should be considered in the calculation. And the vertical displacement of the steel frame structure is consistent with the settlement of the foundation rock. At this time, the steel frame is a cantilever beam elastically fixed on the foot-reinforcement bolt and the foundation rock. The basic mechanical calculation system of supporting structure is shown in Fig 12.

According to the condition that the relative rotation angle, the relative horizontal displacement and the relative vertical displacement at the vault section of the steel frame are all 0, the deformation compatibility equation for structural calculation can be obtained as shown in Eq 1:

$$\left\{ \begin{array}{l} X_{1}\delta_{11}+X_{2}\delta_{12}+\Delta_{1\text{p}}+\left(\beta_{1}+\beta_{2}\right)=0\\ X_{1}\delta_{21}+X_{2}\delta_{22}+\Delta_{2p}+\left(\mu_{1}+\mu_{2}\right)\sin\alpha +\\ \left(\nu_{1}+\nu_{2}\right)\cos\alpha +f\left(\beta_{1}+\beta_{2}\right)\sin\alpha +\left(l_{2}\beta_{2}-l_{1}\beta_{1}\right)\cos\alpha =0\\ X_{3}\delta_{33}+\Delta_{3p}-\left(\mu_{1}+\mu_{2}\right)\cos\alpha +\\ \left(\nu_{1}+\nu_{2}\right)\sin\alpha -f\left(\beta_{1}+\beta_{2}\right)\cos\alpha +\left(l_{2}\beta_{2}-l_{1}\beta_{1}\right)\sin\alpha =0\\ \nu_{4}\text{=}-X_{4}/K_{1}A_{1}\\ \nu_{5}\text{=}X_{5}/K_{2}A_{2} \end{array}\right.$$ (1)

Where: *δ*_11_, *δ*_12_, *δ*_21_, *δ*_22_ and *δ*_33_ are the unit displacements along the above three force directions under the action of *X*_1_, *X*_2_ and *X*_3_ when the steel frame arch foot is fixed respectively. It can be concluded from the structural characteristics of the model: *δ*_13_ = *δ*_31_ = *δ*_23_ = *δ*_32_ = 0. Δ_1p_, Δ_2p_ and Δ_3p_ are the unit displacements of the vault section along the directions of above three force *X*_1_, *X*_2_ and *X*_3_ under the action of surrounding rock load when the arch foot is rigidly fixed respectively, *ν*_4_ and *ν*_5_ are the vertical displacements of the foundation rock at the bottom of the primary support structure and the middle wall structure under the structure load respectively; *A*_1_ and *A*_2_ are the cross-sectional areas of primary support steel frame and middle partition steel frame respectively; The initial support and the arch foot displacement of the steel frame of the middle partition wall are respectively *β*_1_, *ν*_1_, *μ*_1_ and *β*_2_, *ν*_2_, *μ*_2_, which can be calculated according to Eq 2:

$$\begin{array}{l} \beta_{\text{1}}=X_{1}\beta_{1\text{1}}+X_{2}\left[\beta_{2\text{1}}+\left(f-l_{1}/\tan\alpha \right)\sin\alpha \beta_{11}\right]+X_{3}\left[\beta_{31}-\left(l_{1}+l_{2}+l_{4}\right)\sin\alpha \beta_{11}\right]+X_{4}\beta_{4}+\beta_{p1}\\ \beta_{2}=X_{1}\beta_{12}+X_{2}\left[\beta_{22}+\left(f/\sin\alpha -l_{4}\cos\alpha \right)\beta_{12}\right]+X_{3}\left(\beta_{32}-l_{4}\sin\alpha \beta_{12}\right)+X_{5}\beta_{5}+\beta_{p2}\\ \mu_{\text{1}}=X_{1}\mu_{1\text{1}}+X_{2}\left[\mu_{2\text{1}}+\left(f-l_{1}/\tan\alpha \right)\sin\alpha \mu_{11}\right]+X_{3}\left[\mu_{31}-\left(l_{1}+l_{2}+l_{4}\right)\sin\alpha \mu_{11}\right]+X_{4}\mu_{4}+\mu_{p1}\\ \mu_{2}=X_{1}\mu_{12}+X_{2}\left[\mu_{22}+\left(f/\sin\alpha -l_{4}\cos\alpha \right)\mu_{12}\right]+X_{3}\left(\mu_{32}-l_{4}\sin\alpha \mu_{12}\right)+X_{5}\mu_{5}+\mu_{p2}\\ \nu_{\text{1}}=X_{1}\nu_{1\text{1}}+X_{2}\left[\nu_{2\text{1}}+\left(f-l_{1}/\tan\alpha \right)\sin\alpha \nu_{11}\right]+X_{3}\left[\nu_{31}-\left(l_{1}+l_{2}+l_{4}\right)\sin\alpha \nu_{11}\right]+X_{4}\nu_{4}+\nu_{p1}\\ \nu_{2}=X_{1}\nu_{12}+X_{2}\left[\nu_{22}+\left(f/\sin\alpha -l_{4}\cos\alpha \right)\nu_{12}\right]+X_{3}\left(\nu_{32}-l_{4}\sin\alpha \nu_{12}\right)+X_{5}\nu_{5}+\nu_{p2} \end{array}\}$$ (2)

Where: *β*_11_, *β*_12_, *μ*_11_, *μ*_12_, *ν*_11_ and *ν*_12_ are the rotation angle, horizontal displacement and vertical displacement of the arch foot section of the primary support and middle wall steel frame under the unit bending moment; *β*_21_, *β*_22_, *μ*_21_, *μ*_22_, *ν*_21_ and *ν*_22_ are the rotation angle, horizontal displacement and vertical displacement of the arch foot section of the primary support and middle wall steel frame under the unit horizontal force; *β*_31_, *β*_32_, *μ*_31_, *μ*_32_, *ν*_31_ and *ν*_32_ are the rotation angle, horizontal displacement and vertical displacement of the arch foot section of the primary support and middle wall steel frame under the unit vertical force; *β*_p1_, *β*_p2_, *μ*_p1_, *μ*_p2_, *ν*_p1_ and *ν*_p2_ are the rotation angle, horizontal displacement and vertical displacement of the arch foot section of the primary support and middle wall steel frame under the surrounding rock load. It can be calculated according to the following Eqs 3 and 4 [16].

$$\left\{ \begin{array}{l} \beta_{11}=\frac{4\alpha_{1}{}^{3}}{KD_{1}}\\ \mu_{11}=\beta_{21}=\frac{2\alpha_{1}{}^{2}}{KD_{1}}\sin\theta \\ \mu_{21}=\frac{2\alpha_{1}}{KD_{1}}\sin^{2}\theta \\ \nu_{11}=\beta_{31}=-\frac{2\alpha_{1}{}^{2}}{KD_{1}}\cos\theta \\ \nu_{21}=\mu_{31}=-\frac{\alpha_{1}}{KD_{1}}\sin2\theta \\ \nu_{31}=\frac{2\alpha_{1}}{KD_{1}}\cos^{2}\theta \\ \beta_{p1}=\frac{2\alpha_{1}{}^{2}}{KD_{1}}\left(2\alpha_{1}M_{p1}+Q_{p1}\right)\\ \mu_{p1}=\frac{2\alpha_{1}}{KD_{1}}\left(\alpha_{1}M_{p1}+Q_{p1}\right)\sin\theta \\ \nu_{p1}=-\frac{2\alpha_{1}}{KD_{1}}\left(\alpha M_{p1}+Q_{p1}\right)\cos\theta \end{array}\right.$$ (3)

$$\left\{ \begin{array}{l} \beta_{12}=\frac{4\alpha_{2}{}^{3}}{KD_{2}}\\ \mu_{12}=\beta_{22}=\frac{2\alpha_{2}{}^{2}}{KD_{2}}\sin\theta \\ \mu_{22}=\frac{2\alpha_{2}}{KD_{2}}\sin^{2}\theta \\ \nu_{12}=\beta_{32}=\frac{2\alpha_{2}{}^{2}}{KD_{2}}\cos\theta \\ \nu_{22}=\mu_{32}=\frac{\alpha_{2}}{KD_{2}}\sin2\theta \\ \nu_{32}=\frac{2\alpha_{2}}{KD_{2}}\cos^{2}\theta \\ \beta_{p2}=\frac{2\alpha_{2}{}^{2}}{KD_{2}}\left(2\alpha_{2}M_{p2}+Q_{p2}\right)\\ \mu_{p2}=\frac{2\alpha_{2}}{KD_{2}}\left(\alpha_{2}M_{p2}+Q_{p2}\right)\sin\theta \\ \;\nu_{p2}=\frac{2\alpha_{2}}{KD_{2}}\left(\alpha_{2}M_{p2}+Q_{p2}\right)\cos\theta \end{array}\right.$$ (4)

The *M*_pi_ and *Q*_pi_ can be obtained from Eq (5):

$$\left\{ \begin{array}{l} M_{p1}=-q\cdot l_{1}\cdot \frac{1}{2}l_{1}-e_{1}\cdot f\cdot \frac{1}{2}f-\int_{0}^{f}\frac{x}{f}\left(e_{2}-e_{1}\right)\left(f-x\right)dx\\ Q_{p1}=-ql_{1}\cos\theta -\left[e_{1}\cdot f+\int_{0}^{f}\frac{x}{f}\left(e_{2}-e_{1}\right)dx\right]\sin\theta \\ M_{p2}=-q\cdot l_{2}\cdot \frac{1}{2}l_{2}-e_{1}\cdot f\cdot \frac{1}{2}f-\int_{0}^{f}\frac{x}{f}\left(e_{2}-e_{1}\right)\left(f-x\right)dx\\ Q_{p2}=-ql_{2}\cos\theta -\left[e_{1}\cdot f+\int_{0}^{f}\frac{x}{f}\left(e_{2}-e_{1}\right)dx\right]\sin\theta \end{array}\right.$$ (5)

According to the basic mechanical calculation system of supporting structure in Fig 12, Unit displacement of steel arch at vault *δ*_11_, *δ*_12_, *δ*_21_, *δ*_22_ and *δ*_33_ can be calculated according to Eqs 6–9:

$$\delta_{\text{11}}=\frac{1}{E_{s}I_{s}}\int_{0}^{\varphi_{m1}}R_{1}d\theta_{1}+\frac{1}{E_{x}I_{x}}\int_{0}^{\varphi_{m2}}R_{2}ds=\frac{R_{1}\varphi_{m1}}{E_{s}I_{s}}+\frac{R_{2}\varphi_{m2}}{E_{x}I_{x}}$$ (6)

$$\begin{array}{l} \delta_{12}=\delta_{21}=\frac{1}{E_{s}I_{s}}\int_{0}^{\varphi_{m1}}\left(R_{1}-R_{1}\cos\theta_{1}\right)R_{1}d\theta_{1}+\frac{1}{E_{x}I_{x}}\int_{0}^{\varphi_{m2}}\left(R_{2}-R_{2}\cos\theta_{2}\right)R_{2}d\theta_{2}\\ \;=\frac{R_{1}{}^{2}\left(\varphi_{m1}-\sin\varphi_{m1}\right)}{E_{s}I_{s}}+\frac{R_{2}{}^{2}\left(\varphi_{m2}-\sin\varphi_{m2}\right)}{E_{x}I_{x}} \end{array}$$ (7)

$$\begin{array}{l} \delta_{22}=\frac{1}{E_{s}I_{s}}{\int_{0}^{\varphi_{m1}}\left(R_{1}-R_{1}\cos\theta_{1}\right)}^{2}R_{1}d\theta_{1}+\frac{1}{E_{x}I_{x}}{\int_{0}^{\varphi_{m2}}\left(R_{2}-R_{2}\cos\theta_{2}\right)}^{2}R_{2}d\theta_{2}\\ \;=\frac{R_{1}{}^{3}\left(3\varphi_{m1}-4\sin\varphi_{m1}+\frac{1}{2}\sin2\varphi_{m1}\right)}{2E_{s}I_{s}}+\frac{R_{2}{}^{3}\left(3\varphi_{m2}-4\sin\varphi_{m2}+\frac{1}{2}\sin2\varphi_{m2}\right)}{2E_{x}I_{x}} \end{array}$$ (8)

$$\begin{array}{l} \delta_{33}=\frac{1}{E_{s}I_{s}}\int_{0}^{\varphi_{m1}}{\left(R_{1}\sin\theta_{1}\right)}^{2}R_{1}d\theta_{1}+\frac{1}{E_{x}I_{x}}\int_{0}^{\varphi_{m2}}{\left(R_{2}\sin\theta_{2}\right)}^{2}R_{2}d\theta_{2}\\ \;=\frac{R_{1}{}^{3}}{2E_{s}I_{s}}\left(\varphi_{m1}-\frac{1}{2}\sin2\varphi_{m1}\right)+\frac{R_{2}{}^{3}}{2E_{x}I_{x}}\left(\varphi_{m2}-\frac{1}{2}\sin2\varphi_{m2}\right) \end{array}$$ (9)

According to the basic mechanical calculation system of supporting structure in Fig 12, displacement of vault under surrounding rock load Δ_1p_, Δ_2p_ and Δ_3p_ can be calculated according to Eqs 10–12:

$$\begin{array}{l} \Delta_{1p}=\int \frac{M_{p}}{E_{s}I_{s}}ds=-\int_{0}^{\varphi_{m1}}\frac{{\left[R_{1}\sin\theta_{1}\sin\alpha -\left(R_{1}-R_{1}\cos\theta_{1}\right)\cos\alpha \right]}^{2}qR_{1}}{2E_{s}I_{s}}d\theta_{1}\\ \;-\int_{0}^{\varphi_{m1}}\frac{{\left[R_{1}\sin\theta_{1}\cos\alpha +\left(R_{1}-R_{1}\cos\theta_{1}\right)\sin\alpha \right]}^{2}\cdot e_{1}R_{1}}{2E_{s}I_{s}}d\theta_{1}\\ \;-\int_{0}^{\varphi_{m1}}\frac{{\left[R_{1}\sin\theta_{1}\cos\alpha +\left(R_{1}-R_{1}\cos\theta_{1}\right)\sin\alpha \right]}^{3}\cdot \left(e_{2}-e_{1}\right)\frac{1}{3}R_{1}}{2E_{s}I_{s}f}d\theta_{1}\\ \;-\int_{0}^{\varphi_{m2}}\frac{{\left[R_{2}\sin\theta_{2}\sin\alpha -\left(R_{2}-R_{2}\cos\theta_{2}\right)\cos\alpha \right]}^{2}qR_{2}}{2E_{x}I_{x}}d\theta_{2}\\ \;-\int_{0}^{\varphi_{m2}}\frac{{\left[R_{2}\sin\theta_{2}\cos\alpha +\left(R_{2}-R_{2}\cos\theta_{2}\right)\sin\alpha \right]}^{2}\cdot e_{1}R_{2}}{2E_{x}I_{x}}d\theta_{2}\\ \;-\int_{0}^{\varphi_{m2}}\frac{{\left[R_{2}\sin\theta_{2}\cos\alpha +\left(R_{2}-R_{2}\cos\theta_{2}\right)\sin\alpha \right]}^{3}\cdot \left(e_{2}-e_{1}\right)\frac{1}{3}R_{2}}{2E_{x}I_{x}f}d\theta_{2}\\ \; \end{array}$$ (10)

$$\begin{array}{l} \Delta_{2p}=\int \frac{yM_{p}}{E_{s}I_{s}}ds=-\int_{0}^{\varphi_{m1}}\frac{\left(R_{1}-R_{1}\cos\theta_{1}\right){\left[R_{1}\sin\theta_{1}\sin\alpha -\left(R_{1}-R_{1}\cos\theta_{1}\right)\cos\alpha \right]}^{2}qR_{1}}{2E_{s}I_{s}}d\theta_{1}\\ \;-\int_{0}^{\varphi_{m1}}\frac{\left(R_{1}-R_{1}\cos\theta_{1}\right){\left[R_{1}\sin\theta_{1}\cos\alpha +\left(R_{1}-R_{1}\cos\theta_{1}\right)\sin\alpha \right]}^{2}\cdot e_{1}R_{1}}{2E_{s}I_{s}}d\theta_{1}\\ \;-\int_{0}^{\varphi_{m1}}\frac{\left(R_{1}-R_{1}\cos\theta_{1}\right){\left[R_{1}\sin\theta_{1}\cos\alpha +\left(R_{1}-R_{1}\cos\theta_{1}\right)\sin\alpha \right]}^{3}\cdot \left(e_{2}-e_{1}\right)\frac{1}{3}R_{1}}{2E_{s}I_{s}f}d\theta_{1}\\ \;-\int_{0}^{\varphi_{m2}}\frac{\left(R_{2}-R_{2}\cos\theta_{2}\right){\left[R_{2}\sin\theta_{2}\sin\alpha -\left(R_{2}-R_{2}\cos\theta_{2}\right)\cos\alpha \right]}^{2}qR_{2}}{2E_{x}I_{x}}d\theta_{2}\\ \;-\int_{0}^{\varphi_{m2}}\frac{\left(R_{2}-R_{2}\cos\theta_{2}\right){\left[R_{2}\sin\theta_{2}\cos\alpha +\left(R_{2}-R_{2}\cos\theta_{2}\right)\sin\alpha \right]}^{2}\cdot e_{1}R_{2}}{2E_{x}I_{x}}d\theta_{2}\\ \;-\int_{0}^{\varphi_{m2}}\frac{\left(R_{2}-R_{2}\cos\theta_{2}\right){\left[R_{2}\sin\theta_{2}\cos\alpha +\left(R_{2}-R_{2}\cos\theta_{2}\right)\sin\alpha \right]}^{3}\cdot \left(e_{2}-e_{1}\right)\frac{1}{3}R_{2}}{2E_{x}I_{x}f}d\theta_{2}\\ \; \end{array}$$ (11)

$$\begin{array}{l} \Delta_{3p}=\int \frac{xM_{p}}{E_{s}I_{s}}ds=\int_{0}^{\varphi_{m1}}\frac{R_{1}\sin\theta_{1}{\left[R_{1}\sin\theta_{1}\sin\alpha -\left(R_{1}-R_{1}\cos\theta_{1}\right)\cos\alpha \right]}^{2}qR_{1}}{2E_{s}I_{s}}d\theta_{1}\\ \;+\int_{0}^{\varphi_{m1}}\frac{R_{1}\sin\theta_{1}{\left[R_{1}\sin\theta_{1}\cos\alpha +\left(R_{1}-R_{1}\cos\theta_{1}\right)\sin\alpha \right]}^{2}\cdot e_{1}R_{1}}{2E_{s}I_{s}}d\theta_{1}\\ \;+\int_{0}^{\varphi_{m1}}\frac{R_{1}\sin\theta_{1}{\left[R_{1}\sin\theta_{1}\cos\alpha +\left(R_{1}-R_{1}\cos\theta_{1}\right)\sin\alpha \right]}^{3}\cdot \left(e_{2}-e_{1}\right)\frac{1}{3}R_{1}}{2E_{s}I_{s}f}d\theta_{1}\\ \;+\int_{0}^{\varphi_{m2}}\frac{R_{2}\sin\theta_{2}{\left[R_{2}\sin\theta_{2}\sin\alpha -\left(R_{2}-R_{2}\cos\theta_{2}\right)\cos\alpha \right]}^{2}qR_{2}}{2E_{x}I_{x}}d\theta_{2}\\ \;+\int_{0}^{\varphi_{m2}}\frac{R_{2}\sin\theta_{2}{\left[R_{2}\sin\theta_{2}\cos\alpha +\left(R_{2}-R_{2}\cos\theta_{2}\right)\sin\alpha \right]}^{2}\cdot e_{1}R_{2}}{2E_{x}I_{x}}d\theta_{2}\\ \;+\int_{0}^{\varphi_{m2}}\frac{R_{2}\sin\theta_{2}{\left[R_{2}\sin\theta_{2}\cos\alpha +\left(R_{2}-R_{2}\cos\theta_{2}\right)\sin\alpha \right]}^{3}\cdot \left(e_{2}-e_{1}\right)\frac{1}{3}R_{2}}{2E_{x}I_{x}f}d\theta_{2} \end{array}$$ (12)

Where: *φ*_m1_ is the angle corresponding to the arc of the primary support steel frame; *φ*_m2_ is the angle corresponding to the arc of the middle wall steel frame; *α* is the angle (acute angle) between the radial tangent line and the horizontal direction of the top of the middle wall steel frame; *R*_1_ is the arc radius of the primary support steel frame; *R*_2_ is the arc radius of the middle wall steel frame; *E*_s_*I*_s_ is the compressive stiffness of the primary support steel frame (*N*·m^2^); *E*_*x*_*I*_*x*_ is the compressive stiffness of the middle wall steel frame (*N*·m^2^).

By submitting *β*_ik_ and *μ*_ik_ from Eqs 3 and 4, and the unit displacement *δ*_ik_ and Δ_ip_ at vault in basic structure from Eqs 6–12 to the compatibility equation Eq (1), the axial force of each section of the primary support steel frame and the middle wall steel frame can be obtained, as shown in Eqs 13 and 14.

$$\begin{array}{l} N\left(\theta_{1}\right)=X_{2}\cos\theta_{1}+X_{3}\sin\theta_{1}+\left(\left[R\sin\left(\alpha -\theta_{1}\right)-\left(R\sin\alpha -f\right)\right]/\sin\alpha \right)\cdot \sin\theta_{1}\cdot q\\ \;+\left(R-f/\sin\alpha \right)\cdot \sin\theta_{1}\cdot q-e_{1}\cdot \left(R\sin\alpha -R\sin\left(\alpha -\theta_{1}\right)\right)\cdot \cos\left(\alpha -\theta_{1}\right)\\ \;-\frac{{\left[R\sin\alpha -R\sin\left(\alpha -\theta_{1}\right)\right]}^{2}\cdot \left(e_{2}-e_{1}\right)}{2f}\cdot \cos\left(\alpha -\theta_{1}\right) \end{array}$$ (13)

$$\begin{array}{l} N\left(\theta_{2}\right)=q\cdot \left[R_{2}\cdot \sin\left(\alpha_{1}+\theta_{2}\right)-\left(R_{2}\cos\alpha_{1}-R_{2}\cdot \cos\left(\alpha_{1}+\theta_{2}\right)\right)\cdot \tan\alpha_{1}-\left(R_{2}\cdot \cos\left(\alpha_{1}+\theta_{2}\right)-R_{2}\cos\alpha_{1}+f\right)\cdot \tan\alpha_{1}\right]\cdot \sin\left(\alpha_{1}+\theta_{2}\right)\\ \;-X_{2}\cdot \sin\theta_{2}-\left[e_{1}\cdot \left(R_{2}\cos\alpha_{1}-R_{2}\cdot \cos\left(\alpha_{1}+\theta_{2}\right)\right)+\frac{\left(e_{2}-e_{1}\right)}{2f}\cdot {\left(R_{2}\cos\alpha_{1}-R_{2}\cdot \cos\left(\alpha_{1}+\theta_{2}\right)\right)}^{2}\right]\cdot \cos\left(\alpha_{1}+\theta_{2}\right)-X_{1}\cos\theta_{2} \end{array}$$ (14)

In order to accurately reflect the interaction between surrounding rock and support structure, the surrounding rock pressure used in mechanical calculation is selected according to the measured contact pressure between surrounding rock and support structure in the field test, and the parameters in the above structural calculation process are obtained from field test. In order to verify the feasibility of the mechanical response model of the supporting structure, the five key measuring points of the primary support structure and the temporary middle wall structure in the first three excavation stages are selected as the calculation and checking datum points. The internal force values of the supporting structure calculated by the mechanical model and the field test values are compared and analyzed, and the calculation results show that the errors between the field measured value and the calculated value of the mechanical model is less than 25%, and the results show that they are in good agreement, which can prove that the mechanical calculation model in this paper has strong applicability and high reliability in engineering.

## 6. Parameter analysis of support structure

From the above-mentioned field tests and analysis of structural construction mechanical behavior, it can be concluded that the mechanical and deformation distribution characteristics of the support structure gradually change with the progress of each construction stage, and the excavation of supporting rock in Part Ⅲ leads to sharp changes in the mechanical and deformation distribution of the support structure. With the gradual progress of tunnel excavation, the whole supporting structure bears the gradually changing surrounding rock load. According to the mechanical calculation model, after the excavation of Part Ⅰ, the primary support steel frame is firmly welded with the foot-reinforcement bolt at the bottom, and the load transmitted by the upper steel frame structure is shared by the foot-reinforcement bolt and the bottom foundation rock. After the excavation of Part Ⅱ, the load transmitted by the upper steel frame structure is mainly borne by the foot-reinforcement bolt. Therefore, as a part of the joint bearing structure, the foot-reinforcement bolt bears the load of different levels from the upper support structure. Its mechanical properties and deformation behavior under different structural loads are of great significance to the overall safety and stability of the supporting structure in the process of tunnel construction. Therefore, it is necessary to study the mechanical and deformation distribution of the foot-reinforcement bolt with different structural parameters under structure load with different levels.

According to the mechanical calculation and analysis in literature [35] and this paper, the bending strain of each section of the anchor pipe is calculated as shown in Eqs 15–17:

$$\varepsilon_{b}=\frac{M_{0}e^{-\alpha x}\left[\cos\left(\alpha x\right)+\sin\left(\alpha x\right)\right]+Q_{0}\frac{1}{\alpha }e^{-\alpha x}\sin\left(\alpha x\right)}{I_{x}}\cdot y$$ (15)

$$\begin{array}{l} M_{0}=X_{1}+X_{2}\cdot \left(R-R\cos\varphi_{m1}\right)-X_{3}\cdot R\sin\varphi_{m1}-\frac{1}{2}\cdot {\left[R\sin\varphi_{m1}\sin\alpha -\left(R-R\cos\varphi_{m1}\right)\cos\alpha \right]}^{2}q\\ -\frac{1}{2}\cdot {\left[R\sin\varphi_{m1}\cos\alpha +\left(R-R\cos\varphi_{m1}\right)\sin\alpha \right]}^{2}e_{1}-\frac{1}{6f}\cdot {\left[R\sin\varphi_{m1}\cos\alpha +\left(R-R\cos\varphi_{m1}\right)\sin\alpha \right]}^{3}\left(e_{2}-e_{1}\right) \end{array}$$ (16)

$$Q_{0}=q\cdot l_{1}\cdot \sin\left(\alpha -\varphi_{m1}\right)+X_{3}\cdot \cos\varphi_{m1}+\left(e_{1}\cdot f+e_{2}\cdot f/2\right)\cdot \cos\left(\alpha -\varphi_{m1}\right)-X_{1}\cdot \sin\varphi_{m1}$$ (17)

The axial force of each section of the foot-reinforcement bolt is shown in Eq 18:

$$N\left(x\right)=\left[\begin{array}{l} X_{2}\cos\left(\varphi_{m1}\right)+X_{3}\sin\left(\varphi_{m1}\right)+\left(\left[R\sin\left(\alpha -\varphi_{m1}\right)-\left(R\sin\left(\alpha \right)-f\right)\right]/\sin\left(\alpha \right)\right)\cdot \sin\left(\varphi_{m1}\right)\cdot q\\ +\left(R-f/\sin\left(\alpha \right)\right)\cdot \sin\left(\varphi_{m1}\right)\cdot q-e_{1}\cdot \left(R\sin\left(\alpha \right)-R\sin\left(\alpha -\varphi_{m1}\right)\right)\cdot \cos\left(\alpha -\varphi_{m1}\right)\\ -\frac{{\left[R\sin\left(\alpha \right)-R\sin\left(\alpha -\varphi_{m1}\right)\right]}^{2}\cdot \left(e_{2}-e_{1}\right)}{2f}\cdot \cos\left(\alpha -\varphi_{m1}\right) \end{array}\right]\cdot \left[\frac{1}{\eta }sh\left(\varepsilon x\right)-ch\left(\varepsilon x\right)\right]$$ (18)

**Fig 13:**
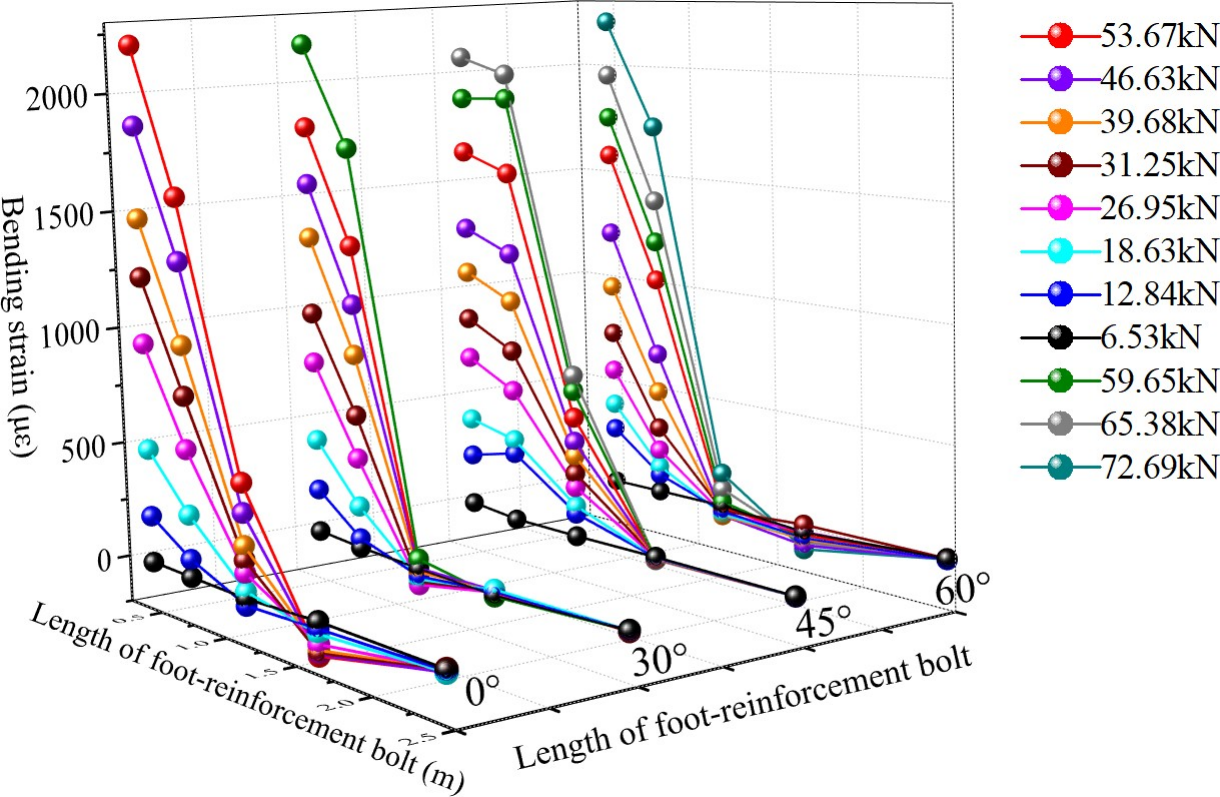
Mechanical properties of foot-reinforcement bolts under different surrounding rock loads.

**Fig 14:**
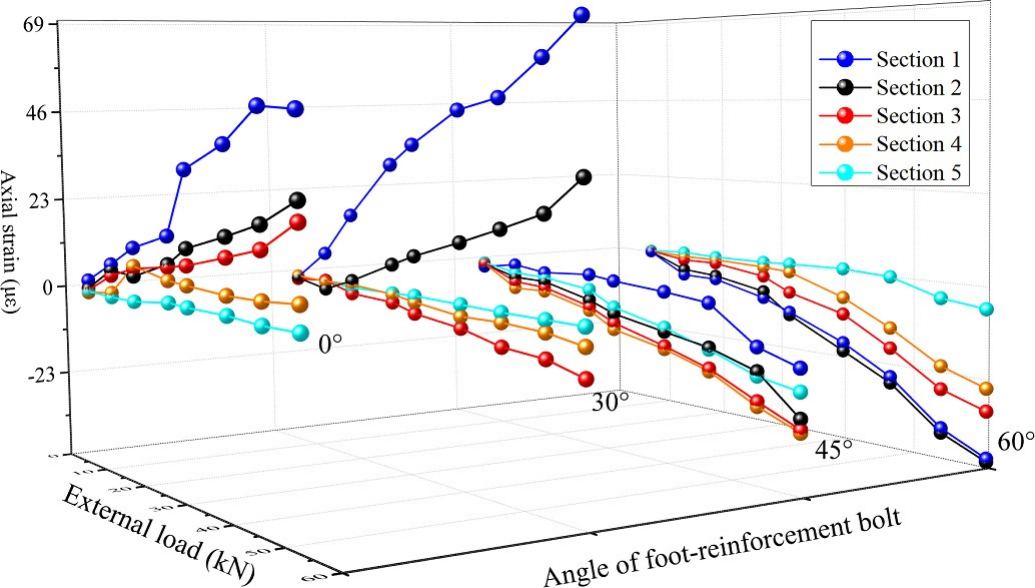
Axial strain distribution of each section of foot-reinforcement bolt under different loads.

The strain of each section of the anchor pipe is actually the combined strain of tension (compression) and bending, and it bears a certain axial compression load as well. The research focuses on the distribution of bending strain and axial strain along the length of the anchor pipe under different levels of load. Mechanical properties of foot-reinforcement bolts with different angles under different surrounding rock loads can be seen in the Fig 13. Axial strain distribution of each section of foot-reinforcement bolt under different loads is as shown in Fig 14.

Under different installation angles, the strain of each section increases with the increase of the external load, which indicates that the anchor pipe can effectively respond to the external load. At the same time, the bending strain of each section of the anchor pipe has a positive correlation with the external load, and the distribution laws of the bending strain along the length of the anchor pipe under the loads with different grades are basically the same. Under different levels of load and different installation angles, the maximum strains of the anchor pipe all appear in Section 1 (near the loading point), and the minimum strain all appears in Section 5 (far away from the loading point). In addition, the bending strain of each measuring point decreases with the increase of the distance from the loading point, and the strain changes slowly along the length of the anchor pipe from section 1 to section 5, and finally tends to zero, which indicates that the end load is gradually transferred to the deep surrounding rock along the anchor pipe. At the same time, the bending strain of section 1 and section 2 is much larger than that of the other three sections, which indicates that the anchor pipe bears the bending load mainly by the bending capacity of the part near the loading point.

Under the same load, the maximum bending strain of anchor pipe decreases with the increase of the setting angle, which is mainly because the shear force at the end of anchor decreases with the increase of the installation angle under the same surrounding rock load. This distribution law shows that increasing the installation angle of the anchor pipe can reduce the bending load on the anchor pipe. The transfer length of the external load varies with the installation angle of the anchor pipe. With the increase of the angle, the section position with strain close to 0 gradually moves to the deeper position of the anchor pipe, which indicates that increasing the installation angle is conducive to transfer external load to the deeper surrounding rock.

The axial strain of each section increases with the increase of load approximately, but the axial strain at 45 degrees first increases and then decreases. Under the same load and installation angle, the axial strain at the end of the anchor pipe is larger than that at the tail, and gradually decreases along the length of anchor pipe, and finally tends to zero at the tail, while for the anchor pipe in 45 degrees, the axial strain of each section first increases and then decreases along the anchor pipe, and reaches the maximum between section 3 and section 4; With the increase of installation angle, the axial strain of each section on the anchor pipe gradually changes from positive strain to negative strain, which indicates that with the increase of angle, the mechanism property of anchor pipe changes from overall tension to partial tension, and finally to overall compression; For the anchor pipe with any angle, the axial strain is small under all levels of load, which indicates that the axial stress of the anchor pipe is small.

## 7. Conclusion

1. The internal force distribution of each position of the supporting structure changes gradually with the excavation of each part. During the excavation of each part, the excavation of Part 1, 2 and 3 has a great influence on the internal force of the supporting structure, and the internal force value of each monitoring position in these three stages reaches 75% of the limit internal force during the whole excavation process. The internal force of the support structure gradually tends to be stable after a slight increase in the excavation of Part 4 and Part 5. The internal force of the supporting structure changes most obviously during the excavation of Part 1, Part 2 and Part 3, and the above three excavation stages are the most unfavorable period for the supporting structure. Compared with other excavation stages, the subsequent excavation of Part 4 and Part 5 has a certain influence on the internal force of the supporting structure.
2. The deformation of different parts of the supporting structure changes gradually with the excavation of each part. On the whole, the vertical displacement of the supporting structure is larger than the horizontal displacement, and the dynamic response of the temporary diaphragm structure during tunnel excavation is shrinkage-expansion-shrinkage-expansion. From the overall deformation distribution, the horizontal deformation and vertical deformation of the support structure mainly occur in the excavation process of Part 1, 2 and 3, and the excavation of Part 4 and 5 has little effect on the deformation response of the structure. The horizontal deformation of the middle diaphragm structure mainly occurs in the excavation process of upper bench, and the excavation of this part has limited influence on the horizontal deformation of the initial support structure. The excavation of the rock mass at the lower bench is the main affecting factor for the horizontal deformation of the primary support arch foot.
3. Under different installation angles, the strain of each section increases with the increase of the end load. In addition, the bending strain of each measuring point decreases with the increase of the distance from the loading point, and the bending strain of section 1 and section 2 is much larger than that of the other three sections. Under the same load, the maximum bending strain of anchor pipe decreases with the increase of the setting angle. The transfer length of the end load varies with the installation angle of the anchor pipe. With the increase of the angle, the section position with strain close to 0 gradually moves to the deeper position of the anchor pipe, which indicates that increasing the installation angle is conducive to transfer end load to the deeper surrounding rock, and the axial strain of each section on the anchor pipe gradually changes from positive strain to negative strain. Under the same setting angle, the axial strain of the same section increases with the increase of load. Under the same load and installation angle, the axial strain at the end of the anchor pipe is larger than that at the tail, and gradually decreases along the length of anchor pipe, and finally tends to zero at the tail except for the situation of 45°. The axial strain of the anchor pipe is small at any angle.

## Supporting information

S1 Data (XLSX)
